# Finite Element Investigation of the Effects of the Low-Frequency Vibration Generated by Vehicle Driving on the Human Lumbar Mechanical Properties

**DOI:** 10.1155/2018/7962414

**Published:** 2018-09-30

**Authors:** Ruo-xun Fan, Jie Liu, Yong-li Li, Jun Liu, Jia-zi Gao

**Affiliations:** ^1^Department of Automotive Engineering, Jilin Institute of Chemical Technology, Jilin 132022, China; ^2^No. 2 Hospital of Jilin University, Jilin University, Changchun 130025, China; ^3^Department of Engineering Mechanics, Jilin University, Changchun 130025, China

## Abstract

Long-term exposure to low-frequency vibration generated by vehicle driving impairs human lumbar spine health. However, few studies have investigated how low-frequency vibration affects human lumbar mechanical properties. This study established a poroelastic finite element model of human lumbar spinal segments L2–L3 to perform time-dependent vibrational simulation analysis and investigated the effects of different vibrational frequencies generated by normal vehicle driving on the lumbar mechanical properties in one hour. Analysis results showed that vibrational load caused more injury to lumbar health than static load, and vibration at the resonant frequency generated the most serious injury. The axial effective stress and the radial displacement in the intervertebral disc, as well as the fluid loss in the nucleus pulposus, increased, whereas the pore pressure in the nucleus pulposus decreased with increased vibrational frequency under the same vibrational time, which may aggravate the injury degree of human lumbar spine. Therefore, long-term driving on a well-paved road also induces negative effects on human lumbar spine health. When driving on a nonpaved road or operating engineering machinery under poor navigating condition, the auto seat transmits relatively high vibrational frequency, which is highly detrimental to the lumbar spine health of a driver.

## 1. Introduction

Long-term exposure to low-frequency vibration impairs human lumbar spine health and increases the risk of low back pain [1, 2]. When the vibrational frequency is close to the natural frequency of human lumbar spine, the rapidly growing stress and deformation in the intervertebral disc aggravates lumbar injury [3]. Under many vibrational environments, low-frequency vibration generated by normal vehicle driving has become an important factor of lumbar injury. With the rapid development of the automotive industry, the risk of lumbar injury for a driver has increased significantly, especially for truck, agricultural machinery, and construction machinery drivers [4, 5].

To determine the lumbar injury mechanism during driving and to explore the protective measures, the effects of low-frequency vibration generated by normal vehicle driving on human lumbar mechanical properties should be investigated primarily. Given that the lumbar mechanical properties simultaneously involve the solid and porous mechanical parameters and load transmission within vertebral bodies is performed internally, measuring lumbar mechanical parameters in vitro may be difficult [6]. Finite element analysis (FEA) can be used as an alternative method to establish the lumbar finite element model (LFEM), simulate the vibrational environment, and predict the lumbar mechanical parameters [7].

Currently, LFEM can be established based on the computed tomography (CT) scan of vertebral bone structure. However, the ligament and the facet are always omitted or replaced with 1D element (such as spring element or rod element) because the soft tissues cannot be developed during scanning [8, 9]. The ligament and the facet play important roles in maintaining lumbar stability and buffering vibration. Hence, omitting or simplifying the ligament and the facet structures in LFEM affects the accuracy of vibrational analysis [10]. Meanwhile, the static response in human lumbar spine was analyzed using LFEM in most studies, which focused on the changes in lumbar mechanical parameters under different quasi-static loading conditions [6, 8]. Few studies investigated the effects of vibrational load on the human lumbar mechanical properties. Even when vibrational condition was simulated, observation of the time-dependent vibrational characteristics between solid and porous mechanical parameters was difficult, because the porous material properties in the established LFEM were not considered. Hence, determining the effects of different vibrational conditions on human lumbar mechanical properties is difficult [11, 12].

Accordingly, this study established a poroelastic finite element model with 3D ligament of human lumbar spinal segments L2–L3 to perform time-dependent vibrational simulation analysis, observed the effects of different vibrational frequencies generated by normal vehicle driving on human lumbar mechanical properties, explained the intrinsic causes of the changes in lumbar mechanical parameters at different vibrational frequencies and times, and explored the lumbar injury mechanism of low-frequency vibration during driving.

## 2. Materials and Methods

### 2.1. Establishment of the LFEM

**Figure 1:**
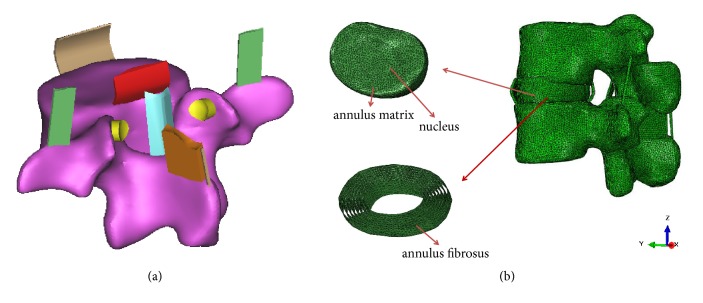
The schematic diagram of human lumbar spine geometric and finite element model. (a) Lumbar geometric model; (b) lumbar finite element model.

The geometrical details of the lumbar spinal segments L2–L3 were obtained from CT scans with a resolution of 1 mm from a healthy young volunteer. The 2D surface of the lumbar structure was reconstructed with MIMICS 10.01 (Materialise, Leuven, Belgium), and the 2D surface was converted to 3D LFEM using tetrahedral element with ABAQUS 6.11 (Simulia, Providence, USA). The ligament and the facet of the lumbar spine, which could not be displayed in the CT scans, were created and assembled between L2 and L3 vertebral bodies by Boolean operation according to the anatomical locations and actual shapes in human lumbar spine (Figure 1(a)). The intervertebral disc structure in the LFEM included annulus ground substance, nucleus pulposus, and annulus fibrosus. Annulus fibrosus were modeled as fiber-reinforced composite, which were embedded in the annulus ground substance in eight layers [6, 17]. The established LFEM can be shown in Figure 1(b).

### 2.2. Lumbar Material Properties

Given that the porous properties in human lumbar spine must be analyzed, biphasic materials, including solid and saturated fluid phases, were assigned to the LFEM. All lumbar spinal structures were assigned biphasic materials, except for the ligaments, facets, and annulus fibrosus, which were merely assigned solid phase [6]. The solid phase in the vertebral bone was assumed to be linear elastic material and the solid phase in the annulus ground substance and nucleus pulposus was represented as Neo-Hookean hyperelastic material [8, 13, 14, 18]. The stiffness of the fibrosus proportionally increased from inside to outside for every two fiber layers, and the proportion varied from 0.65 to 1 [15]. Nonlinear behaviors of the ligaments and the facets were modeled with Neo-Hookean hyperelastic material, and the detailed hyperelastic parameters were fitted from the stress–strain curves in previous experimental study [16]. All fluid material parameters were selected based on references (Table 1).

### 2.3. Boundary and Loading Conditions

The lower surface of the L3 segment in the LFEM was fixed in all directions. The facet contact was set to a surface-to-surface frictionless interaction. Other contacts among the vertebral bodies, intervertebral disc, and ligaments were defined as “TIE” interaction property in ABAQUS [7, 8]. The disc swelling caused by osmotic potential was simulated by a 0.2 MPa boundary pressure on all external surfaces of the vertebral bodies and disc [19]. To investigate the effects of low-frequency vibration on human lumbar mechanical properties, different vibrational frequencies regularly generated by normal vehicle driving must be defined. When driving on a well-paved road, the vibrational frequency of a vehicle transmitted to human lumbar spine is distributed in the range of 1–10 Hz. For 70 kg weight, approximately 750 N of load is compressed on human lumbar spinal segments L2–L3 while sitting on a chair with backrest [20, 21]. To simulate driving for 1 h, 750 N of static compressive load was loaded on the superior surface of L2 in the LFEM under a sinusoidal axial load of ±75 N with different frequencies of 1, 2, 4, 6, 8, and 10 Hz [22].

## 3. Results

### 3.1. Model Validation

**Figure 2:**
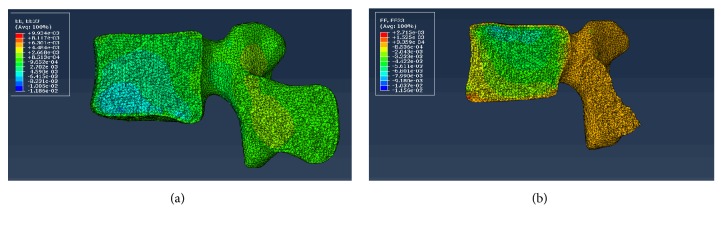
Contour plot of strain on the vertebral bodies of lumbar finite element model under compressive force of 1000 N. (a) L2; (b) L3.

The LFEM established in this study was validated under static and vibrational conditions. To validate the static condition, the strains on the vertebral bodies under a compressive force of 1000 N for 0.5 h were compared with the same condition by in vitro experiment [23]. The experimental results showed that elastic deformations in the anterior, middle, and posterior vertebral bones averaged at 0.694%, 0.301%, and 0.388%, respectively, and creep deformations averaged at 0.433%, 0.163%, and 0.0614%, respectively, thereby showing larger deformations in the anterior than those in the middle and posterior parts. This trend was also observed in our predicted results (Figure 2). Furthermore, the predicted strains in the anterior, middle, and posterior vertebral bones of L2 and L3 were close to the experimental results under the same boundary condition (Table 2).

**Figure 3:**
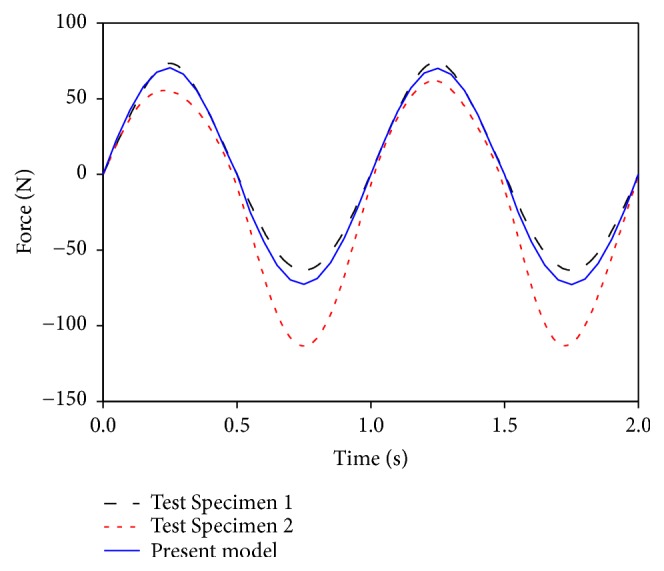
Comparison of the vibrational responses between the established finite element model and the experimental specimens under the sinusoidal anterior–posterior displacement of 0.6 mm at a frequency of 1 Hz.

To validate the vibrational condition, the LFEM was loaded at a sinusoidal anterior–posterior (Y axle) displacement of 0.6 mm at a frequency of 1 Hz, which simulated the vibrational load by the in vitro experiments [4, 24]. Not only was the predicted force–displacement curve consistent with the curve shapes of the two test specimens in the experiment, but the force values were also located in the range of the experimental results, as shown in Figure 3. These validations illustrated the accuracy of the LFEM established in this study.

### 3.2. Effects of Different Vibrational Frequencies on Axial Effective Stress

**Figure 4:**
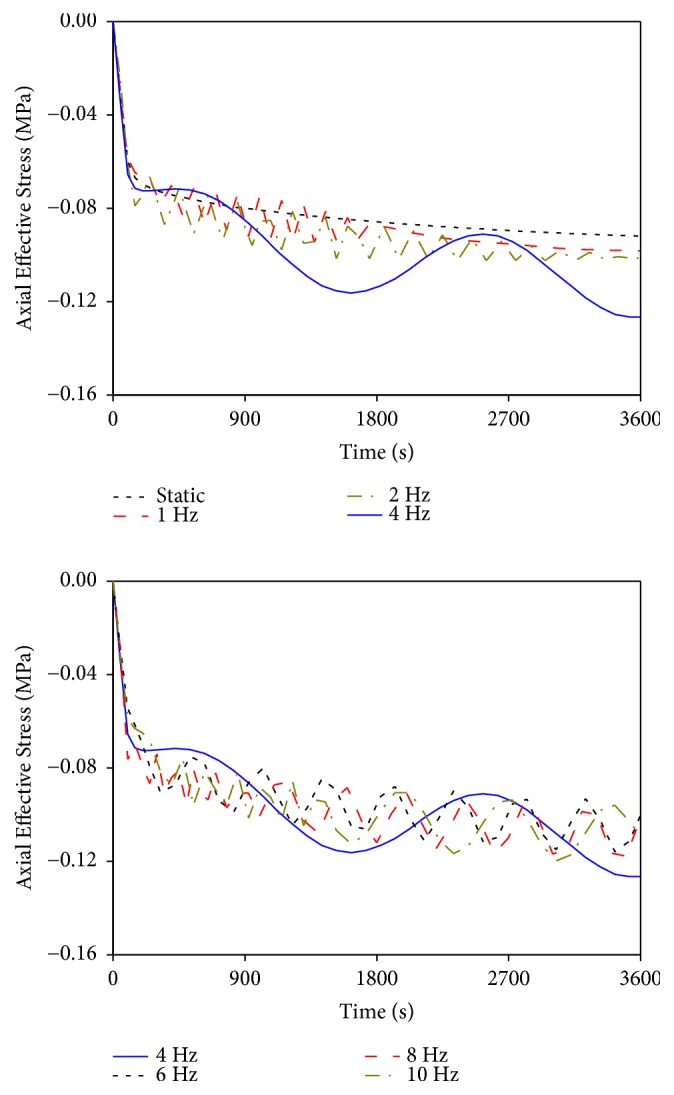
Effects of different vibrational frequencies on axial effective stress.

Axial effective stress is defined as the stress in the vertical direction (Z axle) carried by the solid skeleton in lumbar structure.  Figure 4 shows the effects of different vibrational frequencies on the average axial effective stress in the intervertebral disc with time (average axial effective stress in the intervertebral disc was calculated by dividing the total value of the axial effective stress of all elements by the total number of elements in the intervertebral disc). The axial effective stress increased with time, and the increasing rate varied under different loading conditions. Static load induced relatively less stress and changing rate compared with vibrational load. The numerical fluctuation cycles in the axial effective stress generated by the vibrations at 1 and 2 Hz were short at the first half of loading time (0-0.5 h), and the numerical fluctuation magnitudes gradually disappeared. The curves exhibited a stable linear trend at the second half of loading time (0.5-1 h). The vibrational characteristics of these two curves were similar, and the numerical fluctuation magnitudes in the axial effective stress under 1 and 2 Hz vibrations were less than those generated by other vibrational frequencies. The stress curve generated by the vibration at 4 Hz was different from those in other curves, and the numerical fluctuation magnitude and cycle were evidently higher than those in other vibrational frequencies. Vibrational characteristics at the frequencies of 8 and 10 Hz were similar, and both maximum values in the axial effective stress were higher than the value generated by the vibration at 6 Hz. Additionally, the numerical fluctuation cycles were short at the first half of loading time, and the numerical fluctuation magnitudes were basically unchanged at the second half of loading time for the vibrations at 8 and 10 Hz.

### 3.3. Effects of Different Vibrational Frequencies on Maximum Radial Displacement

**Figure 5:**
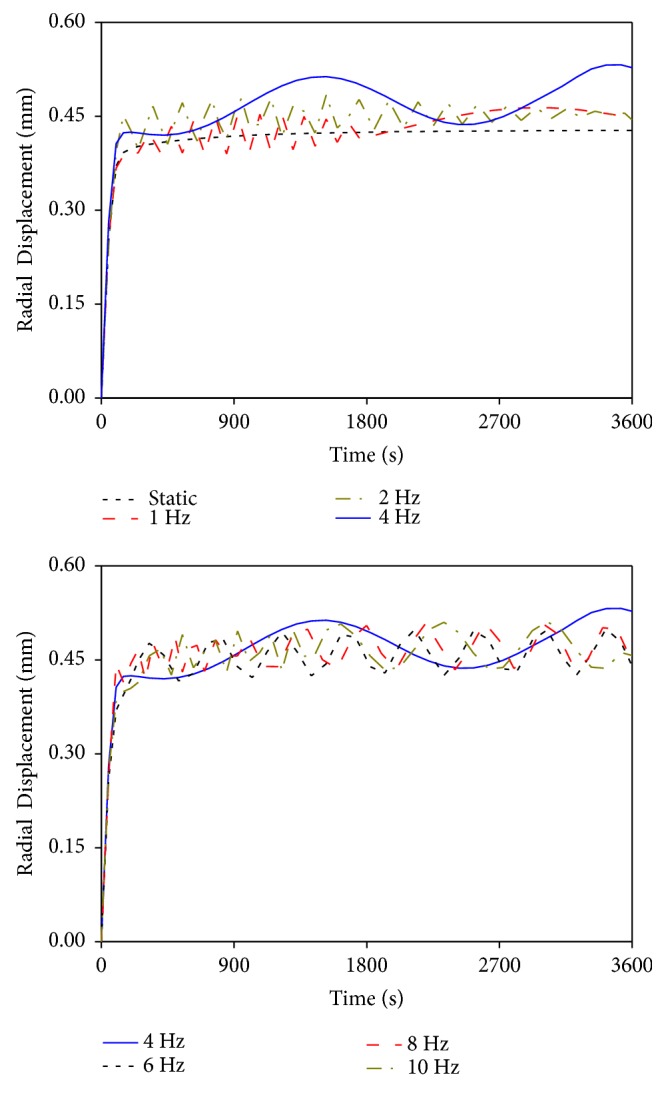
Effects of different vibrational frequencies on maximum radical displacement.

Figure 5 presents the maximum radial displacement in the intervertebral disc as a function of loading time. In the initial stage, each displacement curve was in a linearly rising state, thereby rapidly reaching a high value. All curves except the static load exhibited cyclic change, and the differences in the values of radial displacement were caused by the different numerical fluctuation magnitudes and cycles under a range of vibrational frequencies. The displacement curves under vibrations at 1 and 2 Hz presented nearly no cyclic change at the second half of loading time. The curve at 4 Hz vibration displayed a long numerical fluctuation cycle, and the numerical fluctuation magnitude was large. Therefore, the displacement was significantly higher than those generated by other vibrational frequencies. Under the vibrations at 8 and 10 Hz, the numerical fluctuation cycles during the first half of loading time were short and gradually prolonged with time, and numerical fluctuation magnitudes in the displacement remained nearly the same at the second half of loading time.

### 3.4. Effects of Different Vibrational Frequencies on Fluid Loss

**Figure 6:**
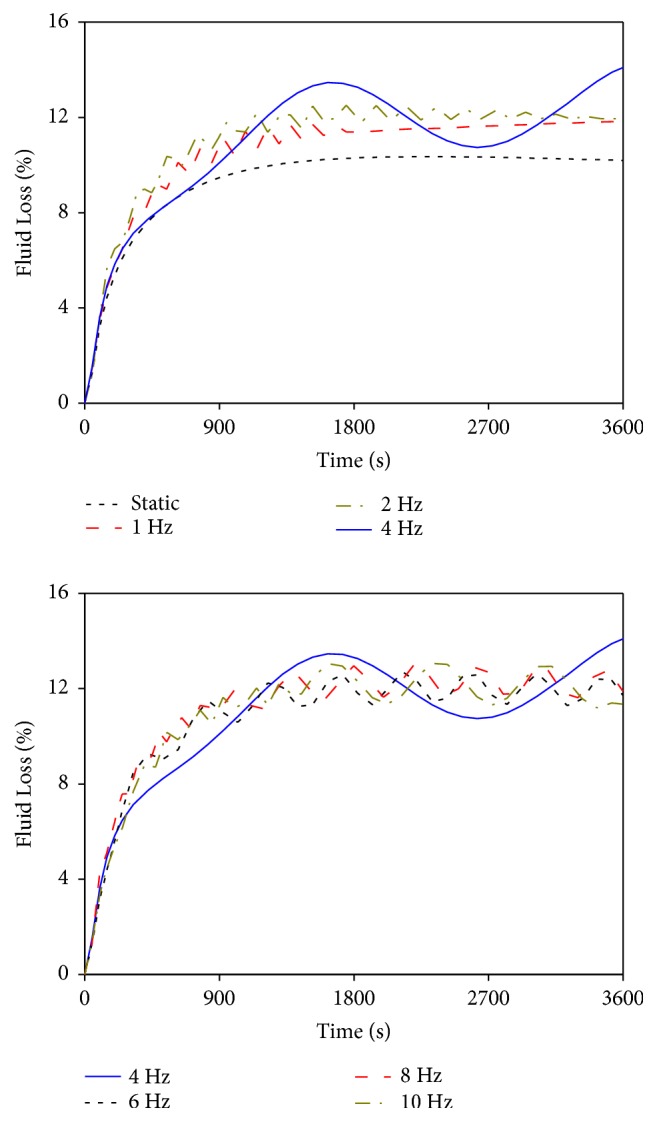
Effects of different vibrational frequencies on fluid loss.

Figure 6 illustrates the changes in the average fluid loss in the nucleus pulposus under static and vibrational loads (average fluid loss in the nucleus pulposus calculated by dividing the total value of the fluid loss of all elements by the total number of elements in the nucleus pulposus). The fluid in the nucleus pulposus exhibited a large loss at the initial stage under different loads, and the decreasing rate rapidly reduced with time. At the second half of loading time, all fluid in the nucleus was no longer being lost with time exclusion of the vibration at 4 Hz. The fluid loss caused by static load was less compared with that by vibrational load. The fluid loss under 1 and 2 Hz vibrations changed rapidly at the first half of loading time but showed a stable linear trend instead of cyclic change at the second half of loading time. Under the vibrations at 6, 8, and 10 Hz, the curves still showed a simple-harmonic cyclic change at the second half of loading time, and the numerical fluctuation magnitudes in fluid loss caused by 8 and 10 Hz were nearly the same and slightly more than the fluid loss caused by the vibration at 6 Hz.

### 3.5. Effects of Different Vibrational Frequencies on Pore Pressure

**Figure 7:**
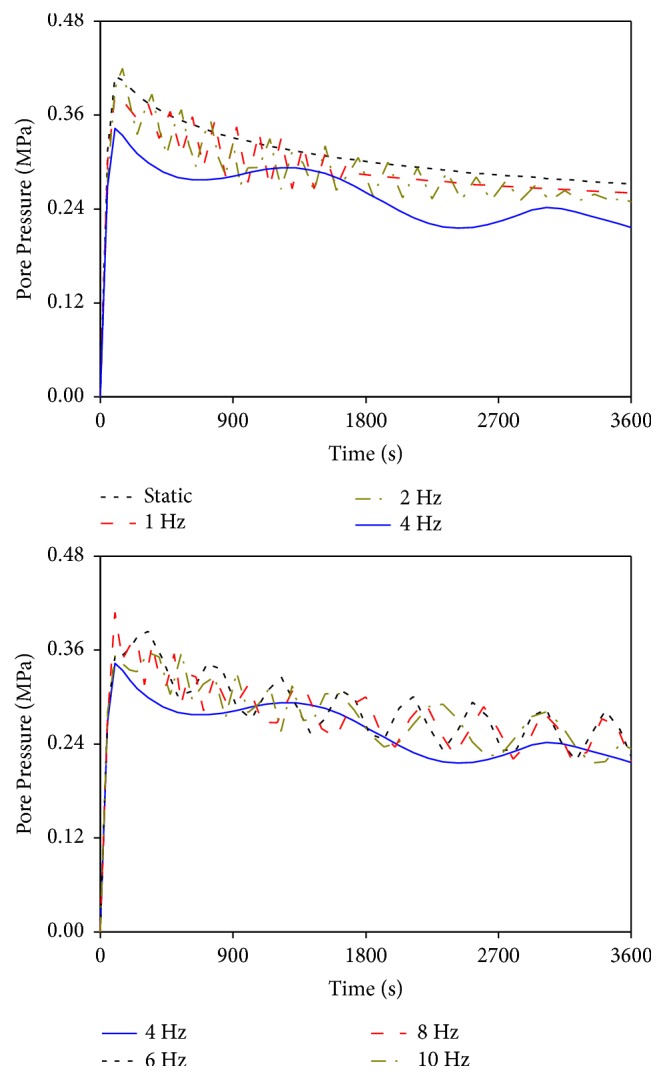
Effects of different vibrational frequencies on pore pressure.

Figure 7 shows the changes in the average pore pressure in the nucleus pulposus under the static and different vibrational loads (average pore pressure in the nucleus pulposus was calculated by dividing the total value of the pore pressure of all elements by the total number of elements in the nucleus pulposus). Under different loads, the peak pore pressures reached at the initial stage of loading and gradually dissipated with time. Static load induced higher pressure and slower decreasing rate than those of vibrational loads, thereby leading to the highest pore pressure at the end of loading. Under the vibrations at 1 and 2 Hz, the numerical fluctuation cycles were short at the first half of loading time and gradually prolonged with time. The pore pressure curve of 4 Hz vibration was different from those of others. Although it showed cyclic change in the value, the numerical fluctuation magnitude of pore pressure varied considerably during two complete cycles, and the decreasing magnitude became large with the cycle. The pore pressure curves caused by the vibrations at 8 and 10 Hz exhibited a similar pattern. The peak pressures in the initial stage and the decreasing rates in the late stage were nearly the same. The pore pressures of the two were both slightly less than the pressure value at 6 Hz vibration under the same vibrational time.

## 4. Discussion

Long-term exposure to low-frequency vibration generated by vehicle driving is detrimental to human lumbar spine health, but the effects of vibrational frequency and duration on the degree of lumbar injury and injury mechanism are unclear [5, 25]. Therefore, this study first established a poroelastic finite element model of human lumbar spinal segments L2–L3 to perform time-dependent vibrational simulation analysis and then discussed the effects of different vibrational frequencies generated by normal vehicle driving on human lumbar mechanical properties.

The simulation results showed that the axial effective stress and the radial displacement in the intervertebral disc, as well as the fluid loss in the nucleus pulposus, increased, whereas the pore pressure in the nucleus pulposus decreased with increased vibrational frequency (excluding the vibration at 4 Hz). High axial effective stress increased the axial deformation in intervertebral disc, thereby resulting in large load-bearing magnitude in annulus ground substance and fibrosus [8]. Excessive radial bulging increased the radial deformation in annulus fibrosus, which may increase the compressive strain and further aggravate fibrosus injury [26]. Pore pressure generated by fluid flow was conducive to resist the external pressure. Given the large fluid content in nucleus pulposus, the load-bearing role of the fluid phase should be prominent. Nevertheless, the excessive fluid loss inside the nucleus resulted in rapid pore pressure dissipation, thereby increasing the load-bearing capacity of the solid skeleton in nucleus pulposus during vibration [19]. Under the combined influences of the above three conditions, nucleus pulposus may break through the damaged annulus fibrosus during compression, which subsequently caused lumbar injury and degeneration [7, 27]. Therefore, increased vibrational frequency generated by normal vehicle driving aggravates human lumbar spine injury.

The simulation results also showed that the responses of the lumbar mechanical parameters during the vibration at 4 Hz were completely different from those in other vibrations. The numerical fluctuation magnitudes of the axial effective stress, radial displacement, and fluid loss were significantly higher than those caused by other vibrational frequencies. Meanwhile, the vibrational curves of the four mechanical parameters at 4 Hz exhibited no stable trend at the last stage of loading, thereby causing continued changes in the values with time. Such changes were different from the stable trends in the vibrational curves at other frequencies. These differences between 4 Hz and other vibrational frequencies may be due to the closeness of the vibrational frequency of 4 Hz to the natural frequency of the LFEM established in this study. When resonance occurred, the lumbar mechanical parameters in the resonant curves only expressed one or two complete numerical fluctuation cycles in an hour. However, vibrations at the other frequencies experienced at least six complete cycles, and the numerical fluctuation cycles of lumbar mechanical parameters gradually shortened as vibrational frequency increased (Figures 4–7). Although the numerical changing cycle in the resonant curve was long, the numerical fluctuation magnitude of the axial effective stress was great in one cycle, within 0.0714–0.126 MPa, thereby directly leading to relatively high stress during vibration (Figure 4). Conversely, the numerical fluctuation magnitude of the pore pressure during the two complete cycles was slight, within 0.236–0.327 MPa, thereby resulting in relatively low pore pressure in the late period (Figure 7). Therefore, occurring resonance may prolong the numerical fluctuation cycle and lead to different variation trends of numerical fluctuation magnitudes for different lumbar mechanical parameters. Meanwhile, the transmission of vibration in human lumbar spine also increased during resonance, which led to detrimental behavior to human lumbar spine health [28, 29]. Therefore, the risk of lumbar injury increases under the vibration at a frequency near the lumbar natural frequency. Pankoke et al. and Li et al. reported in the literature that the vibration-induced resonant frequency of the established human lumbar models was 4 Hz. Magnusson et al. stated that the resonant frequency of the human lumbar spine was in the range of 4–6 Hz according to experimental measurements. All these results were consistent with our predicted resonant frequency using the LFEM [30–32]. This phenomenon not only explained the different changing patterns in the curves between 4 Hz and other vibrational frequencies but also further validated the accuracy of the dynamic FEA in this study.

With the exclusion of the resonant frequency at 4 Hz, the vibrational characteristics in the lumbar mechanical parameters caused by other vibrational frequencies were different and could be divided into two cases. The vibrations at 1 and 2 Hz exhibited a similar pattern, and the vibrations at 6, 8, and 10 Hz exhibited a similar pattern. Under the vibrations at 1 and 2 Hz, all mechanical parameters showed cyclic changes and generated high changing rates during the first half of loading time. Given the well absorbing and damping capacities of lumbar porous structure for low-frequency vibration, the vibrational characteristics of the mechanical parameters at 1 and 2 Hz in the second half of loading time were similar to that of the static load. They reached a linear steady state; that is, the values of the mechanical parameters showed no substantial change with vibrational time. Under the vibrations at 6, 8, and 10 Hz, the vibrational characteristics were nearly the same. The main differences were that the numerical fluctuation magnitudes of the lumbar mechanical parameters at 8 and 10 Hz were slightly higher than those at 6 Hz. The mechanical parameter curves of the three vibrational frequencies also showed cyclic change in the first half of loading time and generated higher numerical fluctuation magnitudes than those of the vibrations at 1 and 2 Hz. Given relatively high vibrational frequency, the lumbar porous structure cannot effectively absorb the vibrational load with short changing cycle, thereby leading to a simple-harmonic cyclic change in mechanical parameters at the last stage of loading. Moreover, a large amount of fluid in the nucleus pulposus was expelled by the vibrations at 6, 8, and 10 Hz, which directly resulted in rapid dissipation of pore pressure. Consequently, the load-bearing magnitude of the solid skeleton in the nucleus pulposus increased. Therefore, vibrations at 6, 8, and 10 Hz may lead to more serious damage to human lumbar spine than those with static and lower vibrational frequencies. Furthermore, the numerical fluctuation cycles and magnitudes of the mechanical parameters for the vibrations at 8 and 10 Hz exhibited few differences. These results indicated that the vibrations at 8 and 10 Hz induced similar degree of injury to human lumbar spine and the numerical fluctuation magnitudes of the mechanical parameters may not change continuously with increased vibrational frequency.

Accordingly, analysis results revealed that static load generated slighter effects on the lumbar mechanical properties than vibrational load. The axial effective stress and the radial displacement in the intervertebral disc, as well as the fluid loss in the nucleus pulposus, increased, whereas the pore pressure in the nucleus pulposus decreased with increased vibrational frequency. These phenomena illustrated that low-frequency vibration generated by vehicle driving was more detrimental to lumbar spine health than static load, and the damage may gradually aggravate as vibrational frequency increased. Therefore, long-term driving on a well-paved road may also have negative effects on human lumbar spine. When driving on a nonpaved road or operating engineering machinery under poor navigating condition, the auto seat will transmit relatively high vibrational frequency, which is highly detrimental to the lumbar spine health of the driver. These results explain why a truck driver is prone to lumbar injury.

The LFEM established in this study presented several limitations. First, the swelling pressure was fixed and uniformly distributed, which was different from strain dependency and nonuniform distribution, as proposed in previous literature [33]. However, within the strain ranges considered, disc swelling pressure varied by 25% or less, and such magnitude may not affect the results significantly. Second, muscular constitution was not simulated in the LFEM. The muscular tissue may not only bear parts of compressive load but also dampen the vibration. Thus, lack of muscular constitution may affect the predicted results. Nonetheless, this study emphasized the effects of vibrational frequencies on the lumbar mechanical properties. The effects of muscular constitution were not considered during analysis. Therefore, the conclusions may not be significantly influenced by the exclusion of muscular constitution [34, 35]. Several limitations were associated with our modeling method, but the model validation, including static and vibrational loading conditions, illustrated the accuracy of the LFEM.

## 5. Conclusion

This study established a poroelastic finite element model of human lumbar spinal segments L2–L3 to perform time-dependent vibrational simulation analysis and then investigated the effects of different vibrational frequencies generated by normal vehicle driving on human lumbar mechanical properties. The analysis results showed that (1) vibrational load induced more damage to human lumbar health than static load; (2) vibration at the resonant frequency generated the most serious injury to human lumbar spine; (3) the axial effective stress and the radial displacement in the intervertebral disc, as well as the fluid loss in the nucleus pulposus, increased, whereas the pore pressure in the nucleus pulposus decreased with increased vibrational frequency, which may aggravate the injury degree of human lumbar spine; and (4) when the vibrational frequency reached a certain degree, the numerical fluctuation magnitudes of the lumbar mechanical parameters were basically unchanged at the late stage of vibration.

## Figures and Tables

**Table 1:** Material properties of the lumbar finite element model.

Structure		Solid phase material			Fluid phase material		References
		Elastic modulus (MPa)		Poisson's ratio	Permeability (m^4^/Ns)	Void ratio	
Cancellous bone	Linear-elastic	100		0.2	1e^−13^	0.4	[8, 13]
Cortical bone	Linear-elastic	10000		0.3	1e^−20^	0.02	[8, 13, 14]
Annulus fibrosus	Linear-elastic	357-550		0.3			[15]
Annulus ground substance	Hyper-elastic	C_10_=0.315	D=0.688		9e^−16^	2.33	[13–15]
Nucleus pulposus	Hyper-elastic	C_10_=0.125	D=2.475		3e^−16^	4	[13–15]
Ligament/Facet	Hyper-elastic	Fitting from previous experiment					[16]

**Table 2:** The predicted elastic and creep strains in different regions of lumbar finite element model under compression.

	Anterior	Middle	Posterior
L2			
Elastic strain	0.689%	0.301%	0.377%
Creep strain	0.497%	0.256%	0.0486%
L3			
Elastic strain	0.671%	0.294%	0.364%
Creep strain	0.485%	0.233%	0.0473%

## Data Availability

The data used to support the findings of this study are available from the corresponding author upon request.
